# Autism Spectrum Disorder: Investigating Predictive Adaptive Behavior Skill Deficits in Young Children

**DOI:** 10.1155/2021/8870461

**Published:** 2021-01-31

**Authors:** Emma Feige, Rhonda Mattingly, Teresa Pitts, Alan F. Smith

**Affiliations:** ^1^Department of Otolaryngology-Head/Neck Surgery-and Communicative Disorders, University of Louisville, Louisville, KY, USA; ^2^Department of Neurological Surgery; Kentucky Spinal Cord Research Centre, University of Louisville, Louisville, KY, USA

## Abstract

Autism spectrum disorder (ASD) is a lifelong neurodevelopmental disorder that consists of difficulties with social communication and language, as well as the presence of restricted and repetitive behaviors. These deficits tend to present in early childhood and usually lead to impairments in functioning across various settings. Moreover, these deficits have been shown to negatively impact adaptive behavior and functioning. Thus, early diagnosis and intervention is vital for future success within this population. The purpose of this study was to further examine the subscales that comprise the adaptive behavior section of the Bayley®-III to determine which of the ten subscales are predictive of ASD in young children (i.e., ≤ three years of age). A retrospective file review of 273 children participating in Kentucky's early intervention program, First Steps, was completed. The children ranged in age from 18 to 35 months. A binary logistic regression was used to assess the subscales that comprise the adaptive behavior of the section of the Bayley®-III to determine which of the ten subscales are predictive of ASD in young children (i.e., ≤ three years of age). The results indicated that individual lower raw scores in communication, community use, functional preacademics, home living, health and safety, leisure, self-care, self-direction, and social subscales were predictive of an autism diagnosis.

## 1. Introduction

Autism spectrum disorder (ASD) is a lifelong neurodevelopmental disorder that consists of deficits in social communication and language, as well as the presence of restricted and repetitive behaviors [1, 2]. ASD is described as a spectrum disorder as it presents differently in each individual. These deficits tend to present in early childhood and usually lead to impairments in functioning across various settings [2].

The Centers for Disease Control and Prevention report that approximately 1 in 59 children are diagnosed with ASD crossing all racial, ethnic, and socioeconomic groups [3]. Previous research has reported a steady increase in the prevalence of ASD over the past 2 decades [4]. Probable reasons for the increase include the “broadening of diagnostic criteria and improved case recognition” [2]. Moreover, symptomology of ASD tends to present differently in males and females [2]. “Camouflaging theory” suggests that females may “mask sociocommunicative impairments due to increased sensitivity to social pressure to fit in, gendered expectations for social behavior, and strengths in some social-communication skills” [5]. This could result in females possibly being “missed by current diagnostic procedures” [5]. Nonetheless, diagnosis of ASD appears to be 4 times more common in males than in females [3].

Secondary to the “heterogeneity of affected individuals and the genetic complexity” of the disorder, it has been difficult to identify the cause(s) of ASD [2]. Previous research has suggested several possible etiologies; however, the literature remains inconclusive [6]. Bölte, Girdler, and Marschik suggest that many genetic and environmental factors and their interactions may contribute to autism phenotypes, but their specific causal mechanisms remain poorly understood. Yates and Le Couteur [2] suggest that significant genetic variations have been found in approximately 10% of individuals diagnosed with ASD. Increased paternal and maternal age has also been associated with higher risk of having a child with autism, possibly due to “de novo spontaneous mutations and/or alterations in genetic imprinting” [7]. Moreover, strong heritability has been linked with ASD as recurrence rates for siblings have been reported to be up to 18.7% [2]. “Research continues to study neurobiological differences in ASD considering variation in neurotransmitters, volumetric and functioning differences of various regions within the brain, but the relevance to clinical practice of most identified abnormalities has not been established” [2].

Environmental factors may also play a role in possible ASD diagnosis. Previous research [8] found that exposure to environmental neurotoxicants during prenatal, perinatal, and postnatal development has been shown to influence the biochemical brain development, resulting in “neurodevelopmental abnormalities that may contribute to ASD.” More specifically, prenatal exposures to “air pollution, heavy metals, pesticides, and toxic substances in consumer products” could bring about atypical brain development, resulting in possible neural pathologies such as ASD [9]. Through growing research, it has become more evident that the etiology associated with ASD is multifactorial with genetic and environmental factors playing a role [7].

The heterogeneity of ASD is evident in the early years of development as well [10, 11]. Kanner first described autism as being one of an “infantile” type, suggesting that the onset of symptoms occurred throughout the early ages of life [7]. Another study examined three possible types/developmental trajectories of ASD in children [12]. These three types include early onset, regression, and plateau [12]. ASD symptoms manifest soon after birth in children with the early onset type, whereas children with the regressive type begin to develop normally until around two years of age proceeded by a regression in development [12]. This regression is most evident in the child's language and social skills [12]. Last, children with the plateau type develop normally until approximately six months of age and cease to make any developmental advances [12]. For example, Rogers [13] describes a halting of development where “babbling was present but did not continue to develop into speech.” Regarding ongoing development and future outcomes, evidence suggests that children who present with the regressive developmental trajectory tend to have more severe deficits across time and in a variety of areas [10].

While the DSM-V provides guidelines and criteria—including severity levels—for diagnosing ASD, it also highlights the fact that symptoms must also be present during early childhood. Under the Individuals with Disabilities Education Act (IDEA), specifically Part C, the law defines the age range for children eligible for early intervention serves as birth to three years of age [14]. The American Speech-Language-Hearing Association defines early intervention as providing families, toddler, and infants who have or are at risk of a developmental delay, disability, or other health condition that inhibits typical development with intervention services [15].

Evidence suggests that the earlier a child receives intervention, the greater the likelihood of an improved developmental trajectory [16]. In general, intensive intervention implemented before age three has been associated with better communicative, academic, and behavioral outcomes at school age [17]. Several studies have concluded that children with autism make greater gains in intervention when it begins earlier, between the ages of two and four, as compared to older children receiving the same interventions, including those with other neurodevelopmental disorders [18]. More recent emerging evidence supports the idea that earlier and more intensive treatment results in more favorable outcomes [19].

Early intervention services often address the needs of children across five developmental areas, including cognitive, motor, social-emotional, communication, and adaptive development [14]. Children referred for early intervention services typically undergo an in-depth evaluation process to assess their therapeutic needs prior to intervention. Various assessment measures may be used during this process with differing requirements from state-to-state. Nonetheless, the assessment process should be comprised of a comprehensive set of activities to (1) identify a child's strengths and weaknesses, (2) address the families concerns and priorities, and (3) develop a plan for ongoing treatment strategies for the child [20, 21].

IDEA requires that the evaluation/assessment be completed using a range of tools in a variety of contexts [14]. The instruments used may include both criterion-referenced and/or standardized properties. One tool, in particular, that is often utilized within early intervention circles is the Bayley Scales of Infant and Toddler Development® (3^rd^ Edition) or the Bayley®-III. The Bayley®-III is a comprehensive assessment tool used to identify developmental issues in early childhood [22].

Previous research has shown that individual lower subscale scores within the cognitive, language, adaptive behavior, and social-emotional developmental domains on the Bayley®-III were predictive of an ASD diagnosis in children three years of age and younger [23]. Due to current literature and ASD diagnostic criteria, this outcome is not surprising with regards to language and social-emotional domains. A direct connection with the cognitive and adaptive behavior sections, however, may be less clear.

Adaptive behavior appears strongly associated with intelligence in neurotypical individuals; however, “cognitively able individuals with ASD fail to acquire adaptive skills at rates corresponding with gains” in intelligence [24]. Moreover, the “gap in daily living skills (i.e., adaptive skills) between children with ASD and typically developing children increased across early childhood” [24] including poorer planning abilities and cognitive flexibility [25]. Nonetheless, a review of the literature examining ASD and adaptive functioning conclude that individuals with ASD tend to present with adaptive functioning difficulties as compared to their same-age peers [24, 26, 27].

Harris and Oakland [28] define adaptive behavior skills as “practical, everyday skills needed to function and meet the demands of one's environment, including the skills necessary to effectively and independently take care of oneself and to interact with other people.” Within the subscale of the adaptive behavior (ADP) skills portion of the Bayley®-III, there are ten subscales. The subscales are comprised of communication (CO), community use (CU), functional preacademics (FA), home living (HL), health and safety (HS), leisure (L), self-care (SC), self-direction (SD), social (S), and motor (M) [22]. These subscales “assess the daily functional skills of a child, measuring what the child actually does, in addition to what he or she may be able to do” [22]. Scores are provided via parent report and are based on the frequency (e.g., is not able, never when needed, sometimes when needed, and always when needed) with which the child performs the behavior when it is needed and without help provided [22].

The purpose of the study was to further examine the subscales that comprise the adaptive behavior section of the Bayley®-III to determine which of the ten subscales are predictive of ASD in young children (i.e., ≤ three years of age). Improved knowledge of the predictive value of each subscale or combination thereof may contribute to an improved understanding of the role adaptive behavior plays in the diagnosis of ASD.

## 2. Methods and Materials

This study utilized a retrospective file review of children (*N* = 273) that participated in Kentucky's early intervention program, First Steps, between 1/1/2012 and 6/1/2019. The sample included children between the ages of 18 and 35 months and comprised 203 males and 70 females. Tabachnick and Fidell [29] recommended a sample size of at least 80, where *N* > 50 + 8*m* (*m* is the number of predictor variables). Moreover, Babyak [30] suggested a minimum sample size of 10–15 observations per predictor variable. Children with and without ASD diagnosis were represented. ASD diagnosis was determined by the intensive level of evaluation (ILE) as completed by the University of Louisville, Weisskopf Child Evaluation Center (WCEC). For the purpose of this study, an ILE is equivalent to a multidisciplinary evaluation that typically involves—in Kentucky—a speech-language pathologist, psychologist, and developmental pediatrician. An occupational therapist may also be involved on a case-by-case basis. Diagnosis is based on majority opinion of the team. Per this study, possible ILE diagnoses included autism with developmental delay or developmental delay. Approval for this study, including the retrospective file review, was granted by the Institutional Review Boards (IRB) of the University of Louisville and the Kentucky Cabinet for Health and Family Services.

The researchers were granted access to the Technology-assisted Observation and Teaming Support (TOTS) database, an electronic record used by the Kentucky Department of Public Health to track children as they are referred, evaluated, and—in some cases—receive services through the early intervention program. The researchers used TOTS to query children referred to—and evaluated by—First Steps between the aforementioned date range. Again, specific interest centered on ASD diagnosis. Demographic information included each child's age (in months) at evaluation and gender. Paper-based files were reviewed at the Kentuckiana Point of Entry office. The Bayley®-III protocols were retrieved from each file (for children diagnosed as having ASD) and randomly for children with developmental delay. The developmental delay sample served as a type of the control group. The raw scores for the ten adaptive behavior subsections and the overall standard deviation scores for the overall adaptive behavior section were anonymously compiled into a Microsoft Excel spreadsheet and then exported to IBM SPSS for Windows, version 25 (IBM Corp., Armonk, N.Y., USA) for statistical analyses. Separate spreadsheets were created for children diagnosed with ASD and those that did not carry the diagnosis. The data were stored on a password protected computer behind a locked door; a master-code was never created. Gender was coded, where 1 = male and 2 = female. ASD diagnosis was coded in the same manner, where 1 = not diagnosed and 2 = diagnosed. No identifying information was recorded.

A binary logistic regression was used to assess the subscales that comprise the adaptive behavior section of the Bayley®-III to determine which of the ten subscales (e.g., communication, community use, functional preacademics, home living, health and safety, leisure, self-care, self-direction, social, and motor) are predictive of ASD in young children (i.e., ≤ three years of age). A binary logistic regression analysis was used, as the criterion variable—ASD diagnosis—is dichotomous [31]. Descriptive statistics, assumption testing, and the results of the logistic regression analyses follow.

## 3. Results

This study comprised a retrospective file review of 273 children in the state of Kentucky: 74.4% (*n* = 203) was male and 25.6% (*n* = 70) was female. The ages ranged from 18 to 35 months (*M* = 24.04, *SD* = 5.30). Forty-eight percent (*n* = 131) of the children were diagnosed with ASD; 52% (*n* = 142) did not have an ASD diagnosis.

Table 1 presents the mean and standard deviations for the ten subscales of the adaptive behavior section of the Bayley®-III [22]. Consistent with regression-based analyses, the ten subscales are referenced as predictor variables. ASD diagnosis served as the criterion variable.

### 3.1. Logistic Regressions are Sensitive to Multicollinearity

“When data are not centered, the regression coefficients that are estimated and tested may be irrelevant and misleading. Centering, thoughtfully done, can diminish the almost inevitable multicollinearity problems in regression, thus increasing both the precision of parameter estimation and the power of statistical testing of those parameters” [32].

As previously suggested, the continuous variables were mean centered by subtracting the mean from the value for each variable. The dichotomous variable—ASD diagnosis—was also centered. This was completed by changing the values of 0 to −0.5 and 1 to 0.5. Variables were centered as a strategy to prevent errors in statistical inference.

A correlation matrix (Pearson) was calculated to assess multicollinearity presence. Mukaka [33] was used to interpret the size of the correlation coefficient. Tabachnick and Fidell [29] suggest that as long as correlation coefficients among independent variables are less than 0.90, multicollinearity is less likely to have occurred. The results are presented in Table 2.

Individual logistic regression analyses were used to examine the relationship between the overall adaptive behavior scale and the associated subscale raw scores with the diagnosis of ASD. Logistic regression allows the use of outcome variables that are categorical and predictor variables that are continuous or categorical. Logistic regression analysis is the most appropriate statistical measure since the criterion variable is dichotomous.  Table 3 shows the results of the logistic regression analysis examining the overall adaptive behavior scale as a predictor of ASD.

The complete results of the logistic regression analyses for the individual subscales that comprise the adaptive behavior scale are presented in Table 4.

### 3.2. Bayley®-III Adaptive Behavior Scale and ASD Diagnosis

Logistic regression—step 1—entered the adaptive behavior scale standard deviation scores as a predictor of ASD diagnosis. The results were statistically significant (odds ratio = 0.12, 95% CI = 0.08–0.20, *p* < 0.001) and explained 53% (Nagelkereke *R*^2^) of the variance of ASD diagnosis. The results suggest that children who receive lower standard deviation scores on the Bayley®-III adaptive behavior scale are more likely to receive an ASD diagnosis than children with higher standard deviation scores.

### 3.3. Bayley®-III Adaptive Behavior Communication Subscale and ASD Diagnosis

Logistic regression—step 1a—entered the adaptive behavior communication subscale raw scores as a predictor of ASD diagnosis. The results were statistically significant (odds ratio = 0.86, 95% CI = 0.83–0.90, *p* < 0.001) and explained 34% (Nagelkereke *R*^2^) of the variance of ASD diagnosis. The results suggest that children who scored lower (raw scores) on the communication subscale on the Bayley®-III adaptive behavior scale are more likely to receive an ASD diagnosis than children with higher communication subscale raw scores.

### 3.4. Bayley®-III Adaptive Behavior Community Use Subscale and ASD Diagnosis

Logistic regression—step 1b—entered the adaptive behavior community use subscale raw scores as a predictor of ASD diagnosis. The results were statistically significant (odds ratio = 0.91, 95% CI = 0.88–0.95, *p* < 0.001) and explained 15% (Nagelkereke *R*^2^) of the variance of ASD diagnosis. The results suggest that children who scored lower (raw scores) on the community use subscale on the Bayley®-III adaptive behavior scale are more likely to receive an ASD diagnosis than children with higher community use subscale raw scores.

### 3.5. Bayley®-III Adaptive Behavior Preacademics Subscale and ASD Diagnosis

Logistic regression—step 1c—entered the adaptive behavior functional preacademics subscale raw scores as a predictor of ASD diagnosis. The results were statistically significant (odds ratio = 0.93, 95% CI = 0.89–0.97, *p* < 0.001) and explained 8% (Nagelkereke *R*^2^) of the variance of ASD diagnosis. The results suggest that children who scored lower (raw scores) on the functional preacademics subscale on the Bayley®-III adaptive behavior scale are more likely to receive an ASD diagnosis than children with higher functional preacademics subscale raw scores.

### 3.6. Bayley®-III Adaptive Behavior Home Living Subscale and ASD Diagnosis

Logistic regression—step 1d—entered the adaptive behavior home living subscale raw scores as a predictor of ASD diagnosis. The results were statistically significant (odds ratio = 0.96, 95% CI = 0.94–0.98, *p* < 0.001) and explained 11% (Nagelkereke *R*^2^) of the variance of ASD diagnosis. The results suggest that children who scored lower (raw scores) on the home living subscale on the Bayley®-III adaptive behavior scale are more likely to receive an ASD diagnosis than children with higher home living subscale raw scores.

### 3.7. Bayley®-III Adaptive Behavior Health and Safety Subscale and ASD Diagnosis

Logistic regression—step 1e—entered the adaptive behavior health and safety subscale raw scores as a predictor of ASD diagnosis. The results were statistically significant (odds ratio = 0.95, 95% CI = 0.93–0.98, *p* < 0.001) and explained 9% (Nagelkereke *R*^2^) of the variance of ASD diagnosis. The results suggest that children who scored lower (raw scores) on the health and safety subscale on the Bayley®-III adaptive behavior scale are more likely to receive an ASD diagnosis than children with higher health and safety subscale raw scores.

### 3.8. Bayley®-III Adaptive Behavior Leisure Subscale and ASD Diagnosis

Logistic regression—step 1f—entered the adaptive behavior leisure subscale raw scores as a predictor of ASD diagnosis. The results were statistically significant (odds ratio = 0.92, 95% CI = 0.89–0.95, *p* < 0.001) and explained 18% (Nagelkereke *R*^2^) of the variance of ASD diagnosis. The results suggest that children who scored lower (raw scores) on the leisure subscale on the Bayley®-III adaptive behavior scale are more likely to receive an ASD diagnosis than children with higher leisure subscale raw scores.

### 3.9. Bayley®-III Adaptive Behavior Self-Care Subscale and ASD Diagnosis

Logistic regression—step 1g—entered the adaptive behavior self-care subscale raw scores as a predictor of ASD diagnosis. The results were statistically significant (odds ratio = 0.93, 95% CI = 0.90–.96, *p* < 0.001) and explained 13% (Nagelkereke *R*^2^) of the variance of ASD diagnosis. The results suggest that children who scored lower (raw scores) on the self-care subscale on the Bayley®-III adaptive behavior scale are more likely to receive an ASD diagnosis than children with higher self-care subscale raw scores.

### 3.10. Bayley®-III Adaptive Behavior Self-Direction Subscale and ASD Diagnosis

Logistic regression—step 1h—entered the adaptive behavior self-direction subscale raw scores as a predictor of ASD diagnosis. The results were statistically significant (odds ratio = 0.95, 95% CI = 0.92–0.97, *p* < 0.001) and explained 10% (Nagelkereke *R*^2^) of the variance of ASD diagnosis. The results suggest that children who scored lower (raw scores) on the self-direction subscale on the Bayley®-III adaptive behavior scale are more likely to receive an ASD diagnosis than children with higher self-direction subscale raw scores.

### 3.11. Bayley®-III Adaptive Behavior Social Subscale and ASD Diagnosis

Logistic regression—step 1i—entered the adaptive behavior social subscale raw scores as a predictor of ASD diagnosis. The results were statistically significant (odds ratio = 0.88, 95% CI = 0.85–0.91, *p* < 0.001) and explained 31% (Nagelkereke *R*^2^) of the variance of ASD diagnosis. The results suggest that children who scored lower (raw scores) on the social subscale on the Bayley®-III adaptive behavior scale are more likely to receive an ASD diagnosis than children with higher social subscale raw scores.

### 3.12. Bayley®-III Adaptive Behavior Motor Subscale and ASD Diagnosis

Logistic regression—step 1j—entered the adaptive behavior motor subscale raw scores as a predictor of ASD diagnosis. The results were not statistically significant (odds ratio = 0.98, 95% CI = 0.96–1.01, *p*=0.14). Although statistical significance was not achieved, the model explained 1% (Nagelkereke *R*^2^) of the variance of ASD diagnosis. Motor subscale raw scores do not seem to vary substantially across ASD diagnostic categories. Per this sample, children with an ASD diagnosis did not appear to have significantly lower motor subscale raw scores than their non-ASD peers.

The intent of this study sought to examine the subscales that comprise the adaptive behavior section of the Bayley®-III to determine which of the ten subscales are predictive of ASD in young children (i.e., ≤ three years of age). The results found that lower standard deviation scores on the adaptive behavior scale on the Bayley®-III was a statistically significant predictor of ASD in young children. Moreover, lower raw scores on the communication, community use, functional preacademics, home living, health and safety, leisure, self-care, self-direction, and social subscales of the adaptive behavior scale of the Bayley®-III were found to be statistically significant predictors of ASD in young children. The communication and social subscales were found to contribute the greatest amount of variance in predicting ASD at 34% and 31%, respectively.

## 4. Discussion and Conclusions

The purpose of this study was to further examine the subscales that comprise the adaptive behavior section of the Bayley®-III to determine which of the subscales are predictive of ASD in young children (i.e., ≤ three years of age) in hope to contribute to the specificity of autism characteristics in early childhood as they relate to adaptive behavior.

The current study examined individual logistic regression analyses which determined that lower standard deviation scores on the adaptive behavior scale on the Bayley®-III was a statistically significant predictor of ASD in young children. Moreover, lower raw scores on the communication, community use, functional preacademics, home living, health and safety, leisure, self-care, self-direction, and social subscales were found to be statistically significant predictors of ASD in young children. The social and communication individual subscale scores contributed the greatest amount of variance when predicting the diagnosis of ASD. As these two deficits are specified within the current diagnostic criteria and there is a vast amount of literature discussing these deficits among the ASD population, these results come as no surprise.

Social and communicative deficits have been diagnostic hallmarks since the first clinical accounts of ASD were recorded [34]. The first clinical accounts were recorded by Dr. Kanner [35], wherein he referenced difficulties with socialization among the observed group of children [34]. Present, one of the first symptoms that is commonly found in children with ASD is their lack of social interaction [36]. Studies examining the relationship between communication skills and corresponding levels of adaptive behavior in individuals with ASD are limited [37]. However, Kjellmer et al. [37] concluded that nonverbal communication skills may be related to severity of autism symptoms as well as adaptive functioning.

The lack of communication skills displayed by children with autism is the greatest cause of concern for parents [17]. As limited communication skills are associated with ASD, these individuals are more likely to display challenging behaviors and/or aggression as this may be their only means of communication, indirectly resulting in increased parental psychological distress [38]. One study examined how parents modified the environment in order to meet the needs of their child with ASD who demonstrated challenging behaviors [39]. The study revealed that parents limited social activities and outings with the child (i.e., shopping and visiting restaurants) [39]. Furthermore, parents avoided taking their child to new and different environments, limiting their exposure into the community [39].

The community use and home living subscales of the Bayley®-III measure a child's ability to participate in activities and interests throughout the community as well as completing household tasks and taking care of personal possessions [22]. According to parent interviews, factors contributing to decreased community and home participation include, but are not limited to, displaying tantrums in community settings as well as demonstrating difficulty with following directions [40].

One study examined participation patterns in preschool children with ASD, specifically within the domains of community mobility and domestic chores [40]. The results indicated that children with ASD participate in significantly fewer activities in all domains compared to typically developing children [40]. Furthermore, the presence of restricted and repetitive behaviors (RRBs) has been shown to set these individuals apart resulting in an increased risk for reduced participation in everyday activities [40]. Liss et al. [41] studied individuals with ASD as they completed the Wisconsin card sorting task (WCST) and observed frequent perseverative behaviors throughout the task that ultimately affected their accuracy and completion. Whereas this task was completed for an experimental purpose, it can emphasize the role repetitive, and perseverative behaviors play on the accuracy and completion of everyday tasks such as domestic chores and self-care routines [42]. Moreover, the presence of restricted and repetitive behaviors can also be linked to deficits in executive function [43]. In a study conducted by Pennington and Ozonoff [44], individuals with autism completed executive functioning tasks with a higher number of perseverative errors as well as exhibited rigid and inflexible problem-solving strategies.

Executive functioning (EF) closely pertains to the cognitive domains of attention, reasoning, and problem-solving [44]. Particularly, “set-shifting and set-maintenance, interference control, inhibition, integration across space and time, planning, and working memory” are that of a few executive functions [44]. Liss et al. [41] further included the processes of “forming abstract concepts, having a flexible sequenced plan of action, focusing and sustaining attention and mental effort, rapidly retrieving relevant information, being able to self-monitor and self-correct as a task is performed, and being able to inhibit impulsive responses” as EF components (p. 261). An individual's level of executive functioning has been shown to correlate with academic skills [41]. Wenz-Gross et al. [45] affirm that EF comprises of “cognitive processes thought to support academic achievement through top-down control of attention and behavior” (p. 2). In general, learning is characterized by the executive functioning tasks of “seeing relationships between pieces of information, identifying central patterns or themes, distinguishing relevant from irrelevant information, and deriving meaning” [44]. As it relates to the present study, the functional preacademic domain within the Bayley®-III assesses preacademic skills such as letter recognition, counting, and drawling simple shapes [22]. The results of this study can be explained by the theory of executive dysfunction, as it is known that individuals with ASD display difficulties with EF as it pertains to academic skills [44]. Conceptual understanding of the main idea or big picture of a topic is often lacking among this group of individuals [44]. Matson et al. [10] state that individuals with ASD exhibit difficulties “executing the mental control necessary for maintaining a problem-solving strategy to obtain a future goal” as well as deficits in cognitive flexibility and planning (p. 445).

The self-care and health and safety domains encompass the skills used in order to complete functional tasks of daily living in addition to the ability to complete those tasks safely and avoid physical dangers [22]. Cavkaytar and Pollard [46] report that many individuals with autism require multiple repetitions of instructions and demonstrate deficits in independently completing daily living skills. One study explored possible reasons for these deficits and included the following: lack of motivation, habits/performance patterns, communication abilities, sensory processing difficulties, and variability in performance [47]. Individuals with autism may not find the value in the self-care task itself nor its outcome and are unlikely to become motivated to finish the task merely to “please an adult or conform to social standards” [47]. With these individuals demonstrating perseverative and stereotyped behaviors, this population tends to stick to strict rituals and routines [1]. Therefore, incorporating new routines to complete tasks of daily living may be difficult to an individual with autism [47]. Additionally, difficulty understanding the task at the hand and the inability for the child to express his/her own needs can affect the completion and/or accuracy of said task [47].

Additionally, it is common for individuals with autism to demonstrate difficulties regarding sensory processing [48]. Sensory difficulties may interfere in with self-care tasks in a number of ways, one of which being unable to teach the child the self-care task [47]. Hand-over-hand assistance will likely be resisted by the child with sensory processing deficits [47]. Last, a variability in performance demonstrated by the child and the inconsistencies of adult responses can influence both “task performance and trajectories of progress” in the realm of completing tasks of daily living [47].

The self-direction and leisure subscales pertain to skills such as self-control, following directions and rules, making choices, playing, and participating in recreational activities within the home [22]. A study conducted by Bachevalier and Loveland [49] found that individuals with ASD demonstrate difficulties with self-regulation of social-emotional behavior. Self-regulation is defined in the aforementioned study as “the ability to select and initiate complex behaviors in response to the specific condition of the social environment” [49]. The ability to self-regulate depends greatly on making inferences about the people and the environment surrounding one's self [49]. With these individuals demonstrating deficits in social communication and social-emotional behavior, self-regulation then becomes difficult [23, 49]. The results of an additional study concluded that children with autism had significant deficits in the “stability of self-regulation and affective expression” as compared to that in individuals with Down syndrome [50]. Furthermore, with measures assessing attention, flexibility, engagement, and goal-directedness during play activities, individuals with ASD demonstrated greater deficits within these realms relative to the group of individuals with Down syndrome [50]. More specifically, the ASD group exhibited difficulties in the ability to sustain attention and concentration to facilitate appropriate play activity [50].

When examining the participation patterns in preschool-aged children with autism, parent interviews revealed children with ASD participate in fewer preschool activities of vigorous leisure [40]. Specific factors affecting decreased participation in leisure include, but are not limited to, the child's inability to follow directions as well as the child's disinterest in the leisure activity [40].

The motor component assesses a child's locomotive abilities as well as his/her ability to manipulate his/her environment [22]. Contrarily, the motor subscale raw score on the Bayley®-III did not significantly contribute to the variance in predicting autism spectrum disorder diagnosis in children ≤ three years of age. Present, the literature is mixed on whether or not motor deficits are a diagnostic characteristic of ASD. Within various studies examining motor coordination, arm movements, gait, and postural stability deficits, individuals with ASD were found to have significant deficits among these motor domains [51]. Likewise, difficulties with postural control, fine and gross motor coordination, and gait abnormalities have been shown to co-occur with an ASD diagnosis [52]. However, in contrast to the aforementioned literature, Ming et al. [53] found no significant association between a diagnosis of ASD and motor deficits. Furthermore, within this study, only 14 children (9%) among the sample group had a history of a gross motor delay, and all 14 of the children achieved gross motor milestones by the enrollment of the study [53]. Additionally, Hanaie et al. [54] investigated the relationship between abnormal corpus callosum connectivity and its effect on sociocommunicative and motor deficits in children with ASD. This study displayed abnormal corpus callosum connectivity relative to sociocommunicative deficits but not as it related to motor deficits in children with ASD [54]. Previously, a study was conducted examining a predictive relationship between the five main developmental domains within the Bayley®-III assessment and a diagnosis of ASD [23]. The results indicated that the motor standard deviation subscale was not significant as an individual predictor of an ASD diagnosis, supportive of the present study's findings [23].

Several factors within this study pose possible limitations. The adaptive behavior portion of the Bayley®-III is assessed based on a questionnaire that is to be filled out by the child's parent, guardian, and/or clinician. This could result in biased data and understanding of the participants. In this case, self-reporting bias may be present [55]. Self-reporting is a common approach utilized by researchers to obtain data and can include questionnaires, surveys, or interviews [55]. Two different types of bias can result from self-reporting—social desirability bias and recall bias [55]. When researchers use self-reporting as a means of data collection, the questions asked may concern private or sensitive topics; in this case, questions were asked regarding the child of the participants' development [55]. Thus, answers to these questions can be “affected by an external bias caused by social desirability or approval” [55]. Furthermore, self-reporting measures may require participants to recall past events resulting in a recall error [55].

Additionally, the evaluation and diagnostic processes for early intervention vary by state. This study obtained files and data from Kentucky's early intervention program—First Steps [56]. Other states may have different protocols and procedures in place when assessing children of three years of age and younger for autism. There are various tools available to early interventionists for the assessment of children of three years of age and younger. This study utilized results from the Bayley®-III due to availability. While this is a popular tool utilized by early interventionists, opportunities for future research can include results from other standardized assessments.

Currently, the literature regarding motor deficits within this population is varied and limited. Future research among this realm will allow for increased specificity in motor characteristics in young children with ASD. As previously mentioned, future research can incorporate other popular assessment tools to examine the different domains and determine if they are predictive of an autism diagnosis. This can allow for a more descriptive analysis of early diagnostic characteristics of autism in young children.

The intent of this study was primarily to contribute to the specificity of early diagnostic characteristics in young children with ASD. More specifically, the study's focus was on the diagnostic characteristics relative to that of adaptive behavior skills. The study encompassed children of three years of age and younger. The findings were consistent with the current body of literature on ASD with respect to deficits in social, communication, functional preacademics, leisure, self-care, self-direction, health and safety, home living, and community use [23, 34, 40, 41, 44, 46, 47, 49].

It is the researchers' belief that with the increased knowledge of ASD characteristics in young children, there will be an increase in a definitive ASD diagnosis at an earlier age. Concurrently, this will allow for these individuals and their families to benefit from early intervention services which have been shown to greatly improve the individual's developmental trajectory. It is our hope that the limited knowledge based on early ASD diagnosis in young children has been increased and the gap in the available literature narrowed.

## Figures and Tables

**Table 1:** Descriptive statistics adaptive behavior subscale raw scores (*N* = 273).

Subscale	*M*	SD
Communication	25.0	10.0
Community use	9.6	8.4
Functional preacademics	6.6	7.9
Home living	22.7	15.3
Health and safety	23.6	11.5
Leisure	28.5	10.2
Self-care	35.8	9.4
Self-direction	29.1	11.0
Social	31.6	10.0
Motor	51.5	11.0

**Table 2:** Pearson product-moment correlation matrix (*N* = 273).

	ADP	CO	CU	FA	HL	HS	LS	SC	SD	SOC
ADP										
CO	*0.57*									
CU	0.47	*0.56*								
FA	0.36	*0.55*	0.49							
HL	*0.51*	*0.59*	**0.72**	0.49						
HS	0.44	*0.62*	*0.61*	0.40	**0.78**					
LS	*0.55*	*0.64*	0.49	0.41	*0.64*	*0.68*				
SC	0.42	*0.58*	0.49	0.26	*0.63*	*0.69*	**0.74**			
SD	0.49	*0.54*	*0.57*	0.29	**0.70**	**0.75**	**0.81**	**0.74**		
SOC	*0.59*	**0.76**	*0.57*	0.44	**0.70**	**0.70**	**0.76**	**0.70**	**0.75**	
MO	0.17	0.40	0.45	0.23	*0.60*	*0.68*	*0.61*	*0.64*	*0.67*	*0.62*

Moderate positive (negative) correlation |*r* = 0.50–0.70| in italics. High positive (negative) correlation |*r* > 0.70| in bold.

**Table 3:** Predicting ASD diagnosis based on adaptive behavior scale standard deviation.

Subscale	Odds ratio	95% (CI)	% variance	*p*
Adaptive behavior	0.12	0.08–0.20	53	<0.001

**Table 4:** Predicting ASD diagnosis based on adaptive behavior subscale raw scores.

Subscale	Odds ratio	95% (CI)	% variance	*p*
Communication	0.86	0.83–0.90	34	<0.001
Community use	0.91	0.88–0.95	15	<0.001
Preacademics	0.93	0.89–0.97	8	<0.001
Home living	0.96	0.94–0.98	11	<0.001
Health/safety	0.95	0.93–0.98	9	<0.001
Leisure	0.92	0.89–0.95	18	<0.001
Self-care	0.93	0.90–0.96	13	<0.001
Self-direction	0.95	0.92–0.97	10	<0.001
Social	0.88	0.85–0.91	31	<0.001
Motor	0.98	0.96–1.01	1	0.14

## Data Availability

The deidentified data used to support the findings of this study are available from the corresponding author upon request.
